# Cellular and Cytokine Responses in Lymph Node Granulomas of Bacillus Calmette Guérin (BCG)-Vaccinated and Non-vaccinated Cross-Breed Calves Naturally Infected With *Mycobacterium bovis*

**DOI:** 10.3389/fvets.2021.698800

**Published:** 2021-09-16

**Authors:** Asegedech Sirak, Begna Tulu, Berecha Bayissa, Balako Gumi, Stefan Berg, Francisco J. Salguero, Gobena Ameni, The ETHICOBOTS Consortium

**Affiliations:** ^1^Animal Health and Zoonotic Research Unit, Aklilu Lemma Institute of Pathobiology, Addis Ababa University, Addis Ababa, Ethiopia; ^2^National Animal Health Diagnostic and Investigation Centre, Sebeta, Ethiopia; ^3^Medical Laboratory Science Department, Bahir Dar University, Bahir Dar, Ethiopia; ^4^Vaccine Production and Drug Formulation Directorate, National Veterinary Institute, Bishoftu, Ethiopia; ^5^Bacteriology Department, Animal and Plant Health Agency, Weybridge, United Kingdom; ^6^Department of Pathology and Infectious Diseases, University of Surrey, Guildford, United Kingdom; ^7^Department of Veterinary Medicine, College of Food and Agriculture, United Arab Emirates University, Al Ain, United Arab Emirates

**Keywords:** BCG vaccination, crossbred calves, immunological response, *Mycobacterium bovis*, natural infection

## Abstract

Local immunological responses at the site of infections, such as at the lymph nodes and lungs, do play a role in containing infection caused by *Mycobacterium bovis* (*M. bovis*). This bovine tuberculosis (bTB) study was conducted to evaluate cellular and cytokine responses in the lymph nodes and lungs of BCG-vaccinated and non-vaccinated calves that were naturally infected with *M. bovis*. Immunohistochemical assays were used for examination of the responses of macrophages, T cells, cytokines and chemical mediators of 40 (22 vaccinated and 18 non-vaccinated) Holstein-Friesian-zebu crossbred calves that were naturally exposed for 1 year to a known bTB positive cattle herd. The incidence rates of bTB visible lesion were 68.2% (15/22) and 89% (16/18) in vaccinated and non-vaccinated calves, respectively. The local responses of CD4^+^ and CD8^+^ T cells, and those of IFN-γ and TNF-α within the lesions, were stronger (*P* < 0.05) in BCG-vaccinated calves than in non-vaccinated calves. However, there was no statistically significant difference between the two groups (*P* > 0.05) in the response of CD68^+^ cells. Thus, the findings of this study indicated stronger responses of a set of immunological cells and markers at the local granulomas of BCG-vaccinated calves than in non-vaccinated calves. Furthermore, BCG vaccination may also play a role in reducing the severity of the gross pathology at the primary site of infection.

## Introduction

Bovine tuberculosis (bTB) is a chronic progressive disease of cattle and other animals that is caused by *Mycobacterium bovis* (*M. bovis*) and is characterized by progressive development of granulomatous lesions, termed as tubercles, in different tissues. BTB poses a major economic problem worldwide costing US$3 billion annually through infecting over 50 million cattle (1). In addition, it causes zoonotic TB in humans particularly in developing countries in Africa, Asia and Latin America (2). The conventional control measures for bTB are based on a test-and-slaughter method, which is less likely to be implemented by the developing countries because of socioeconomic reasons (3). Furthermore, the test-and-slaughter control method have been shown not to be effective in some developed countries, like the United Kingdom and New Zealand, because of the interference of reservoirs of *M. bovis* in infected wildlife (4, 5). Therefore, alternative control methods such as the use of vaccination are required for improved control of bTB.

BCG is the only vaccine approved against human TB (6) and animal TB (7), and it has been used in field studies in cattle yielding variable protective efficacy levels (8–11). Most of these studies evaluated immunological responses induced by BCG vaccination in peripheral blood. In addition, data on the immunological responses induced by BCG-vaccination at the local site of infection (granulomatous lesion) are useful for understanding the roles of different cell types and immunological markers (12–14).

Cell-mediated immune response is crucial in the defense against intracellular bacterial pathogens including mycobacteria (15, 16). Immune cells like CD4, CD8 and γ/δ T cells are shown to be activated when exposed to mycobacterial antigens based on studies conducted in mice, humans, and cattle (17–19). These cells are recruited to the site of infection and are capable of producing various cytokines, including interferon gamma (IFN-γ) and tumor necrosis factor alpha (TNF-α) (20).

BTB granulomas are characterized by different developmental stages that are influenced by the interaction between the host immunological responses and virulence of *M. bovis* isolates (21, 22). The granuloma, the hallmark lesions of TB disease, is characterized by the accumulation of activated macrophages and a variety of other immune cells against the mycobacteria. The formation of the granuloma have long been regarded as the critical host-protective structures that wall off the bacteria, creating an immune microenvironment in which the infection can be controlled. Within the granuloma the mycobacteria are thought to undergo replicative arrest and in response to stress such as hypoxia (23). However, if immunological responses are weakened, the granulomas fail to contain the pathogen, and as a result, the bacilli disseminate to secondary sites of infection, producing multiple granulomas (24). Thus, the interaction between the immune response and the mycobacteria at the local lesion is determining the outcome of the disease. It can also be observed that granuloma protect the mycobacteria from the immune response and is probably responsible for the persistent infection (25, 26). Microscopically, bTB granulomas have been classified into four different stages; stage I being the least severe stage while stage IV being the most severe stage (27, 28). The present study was conducted along a BCG efficacy study (29), to evaluate the cellular and cytokine responses induced by BCG-vaccination in the granulomas of Holstein-Friesian (H-F) and zebu crossbred calves that were naturally exposed to *M. bovis* in Ethiopia.

## Materials and Methods

### Experimental Setting

A BCG efficacy study against bovine TB conducted at the National Animal Health Diagnostic and Investigation Centre (NAHDIC) under natural challenge conditions was utilized for this study to generate immunohistochemical (IHC) information in the same animals. In short, male HF-zebu crossbreed calves were recruited within 2 weeks of age from known bTB free cattle farms as confirmed by the Single Intradermal Comparative Cervical Tuberculin (SICCT) test for which PPD-A and PPD-B were sourced from Thermo Fisher (Lelystad, the Netherlands). Calves were randomly assigned to either the unvaccinated control or the BCG vaccinated group. The latter group was vaccinated subcutaneously with 1 × 10^6^ CFU BCG (InterVax, Canada), a vaccine based on the Russian type of *Mycobacterium bovis* BCG. Six weeks after vaccination, the two groups of calves were introduced into a herd of SICCT test positive cows at a ratio of 2:1 (Experimental calves: Reactor cows). After completion of 12 months exposure, 22 BCG vaccinated and 18 non-vaccinated calves had completed the experiment and they were slaughtered for post mortem examination and collection of tissue samples.

### Sample Collection and Processing

Gross and microscopic pathological examinations as well as immunohistochemical examination were performed by a pathologist who was blinded to the vaccination status of the calves. The pathologist was trained on IHC at the Pathology Laboratory of Surrey University (UK) by Dr. Francisco J. Salguero before performing this experiment. All lymph nodes in the head and neck region (left and right lateral retropharyngeal, left and right medial retropharyngeal, left and right mandibular, and left and right parotid), thoracic (mediastinal, cranial and caudal, right and left bronchial and tracheobronchial) and abdominal (hepatic and mesenteric) cavities, together with the lungs were carefully examined for gross lesions of TB and for mycobacterial culturing by Bayissa et al. (29). For the present study, all TB-like gross lesions were collected into either 10% neutral buffered formalin or zinc salt fixative solution, depending on the type of markers, for further processing for IHC staining.

### Immunohistochemical Staining

All tissue specimens were processed and embedded into paraffin wax in the histopathology laboratory at NAHDIC. Tissues for the investigation of CD68, IFN-γ, TNF-α, and iNOS were fixed in 10% neutral buffered formalin while tissues for the investigation of CD4 and CD8 cells were fixed in zinc salt by following the procedure used previously (30). All formalin fixed tissues were processed within 7 days as previously described (31) whereas zinc solution fixation was carried out for 72 h. Specimens were trimmed and processed using a SHANDON Citadel 1000 tissue processor, dehydrated in six rounds using graded ethyl alcohol (at concentrations 70, 95, 95, and 3 × 100%) to remove excess water from the tissue, and cleared in three passes of xylene to clean alcohol from the tissue and to make the tissue clear/translucent. The tissue samples were then impregnated in melted paraffin (two passes) and embedded using the paraffin wax embedding method by the TEC2900 modular Tissue Embedding Center (Histo-line laboratories). Sections of 4μm were prepared and placed on Vectabond-treated slides (Vector Laboratories, Peterborough, UK).

Lymph nodes and lung tissue sections from each animal were processed by IHC for cell types and chemical mediators using primary antibodies to label CD68, CD4, CD8, IFN-γ, TNF-α, or iNOS. The reagents and antibodies against each of these immunological markers are presented in Table 1 and the staining was conducted as previously described (32). Enzymatic digestion was used as an epitope demasking technique for the formalin fixed material. Trypsin solution was prepared by measuring 0.5 g of trypsin, 0.5 g of chymotrypsin and 1 g of CaCl_2_ and dissolving in 1 L of distilled water. A biotinylated secondary antibody (ABC Vector Elite; Vector Laboratories) was used at 1/200 (Ab + PBS) dilution for CD4 and CD8, and for IFN-γ and CD68 a biotinylated secondary antibody was used by adding 3 drops (135 μl) to 10 ml of normal horse serum (ABC Vector Elite; Vector Laboratories). For detection of TNF-α 1 drop (45 μl) of biotinylated secondary antibody from the ABC kit (ABC Vector Elite; Vector Laboratories) was added to 10 ml of PBS. For iNOS biotinylated Secondary antibody, the same dilution was prepared but instead of normal horse serum, normal goat serum was used from the ABC kit. A horseradish peroxidase-labeled avidin-biotin-complex was used with DAB as a chromogen (brown color) and Mayer's haematoxylin was used as counter-stain.

**Table 1:** Immune markers used for immunohistochemistry.

**Primary antibody**	**Antibody type**	**Dilution**	**Supplier**	**Epitope demasking method**	**Link antibody (dilution)**	**Primary antibody incubation (Temp, Time)**	**Source**	**Buffer**
IFN-γ	Mouse anti bovine IFN-γ (monoclonal)	1/200	BIO-RAD	NA	Horse anti- mouse (1/200)	RT, 1 h	Thermo Scientific	PBS
TNF-α	Mouse anti bovine TNF-α (monoclonal)	1/100	BIO-RAD	Trypsin	Horse anti- mouse (1/200)	RT, 1 h	Thermo Scientific	PBS
CD68	Mouse anti human CD68 (monoclonal)	1/50	BIO-RAD	Trypsin	Horse anti- mouse (1/200)	4°C (Overnight)^*^	Thermo Scientific	PBS
CD4	Mouse anti bovine CD4 (monoclonal)	1/30	BIO-RAD	NA	Goat vs. mouse (1/200)	RT, 1 h	BIO-RAD	PBS
CD8	Mouse anti bovine CD8 (monoclonal)	1/100	BIO-RAD	NA	Goat vs. mouse (1/200)	RT, 1 h	BIO-RAD	PBS
iNOS	Rabbit anti mouse iNOS (polyclonal)	1/500	BIO-RAD	NA	Goat anti-rabbit (1/1,000)	RT, 1 h	Thermo Scientific	PBS

* *The optimization for anti-CD68 primary antibody was done overnight at 4°C*.

*NA, Not Applicable; PBS, Phosphate buffered saline; RT, Room Temperature*.

### Image Analysis

Stained sections were subjected to digital image analysis to ascertain the percentage area of the tissue section positively labeled (brown color) for each marker. Images were captured using light microscopy (Nikon ECLIPSE E200 LED) and digital image analysis software (Nikon-NIS Br, Nikon, Japan). Consecutive sections were used and all granulomas observed within the tissue section were analyzed. The whole area of the granuloma was selected as Region of Interest (ROI), and the area with immunohistochemically-positive reaction within the ROI was calculated by the software after setting the thresholds. The results are expressed as the percentage of positively stain area within the total area of the granuloma. Necrotic or mineralized areas were not included in the ROI to be analyzed as previously described (32).

### Data Analysis

Chi-square test for trend was used to compare the distribution of granulomas within the head and neck, thoracic, and abdominal lymph nodes as well as the lung sections. The presence of the different markers and cytokines were compared between the granulomas of vaccinated and non-vaccinated calves using the Mann-Whitney's *U*-test by Graph Pad Prism 8.0.2.263. This test was used to compare the medians and for all statistical analysis *P* < 0.05 was considered statistically significant.

## Results

### Gross Pathology

BTB visible lesions were detected in 68.0% (15/22) of the BCG vaccinated and 89.0% (16/18) of the unvaccinated calves. Seven calves from the BCG vaccinated group and two calves from the BCG non-vaccinated group were found to be free from bTB visible lesions. Most of the visible lesions were found in the thoracic lymph nodes of both BCG vaccinated and non-vaccinated calves. In addition, the 31 animals with visible lesions were all confirmed by culture to be *M. bovis* infected (29).

### Histopathology

The histopathological examination was used to identify different stages of granulomas of both the 15 BCG-vaccinated and the 16 non-vaccinated calves that had TB lesions in lymph nodes and/or lung tissues. In total, 79 lymph nodes and lung tissues were taken forward and investigated by histopathology and IHC, of which 34 were from BCG-vaccinated and 45 were from non-vaccinated calves. The majority of granulomas were found within the thoracic lymph nodes, with more stage IV granulomas present in the control group when compared to the BCG vaccinated (*P* < 0.05). A similar trend was observed for the lung sections, with many granulomas observed (all stages) in the control group while only few stage II and III granulomas were found in animals from the BCG vaccinated group. In the head lymph nodes, only stage I granulomas were found in the BCG vaccinated group while granulomas of all developmental stages were found in the control group (Supplementary Table 1). In contrast, more granulomas were observed in the abdominal cavity lymph nodes from BCG vaccinated animals, including several stage IV granulomas, while control animals only showed a few stage I and II granulomas (*P* < 0.05).

### Immunohistochemistry

**Figure 1:**
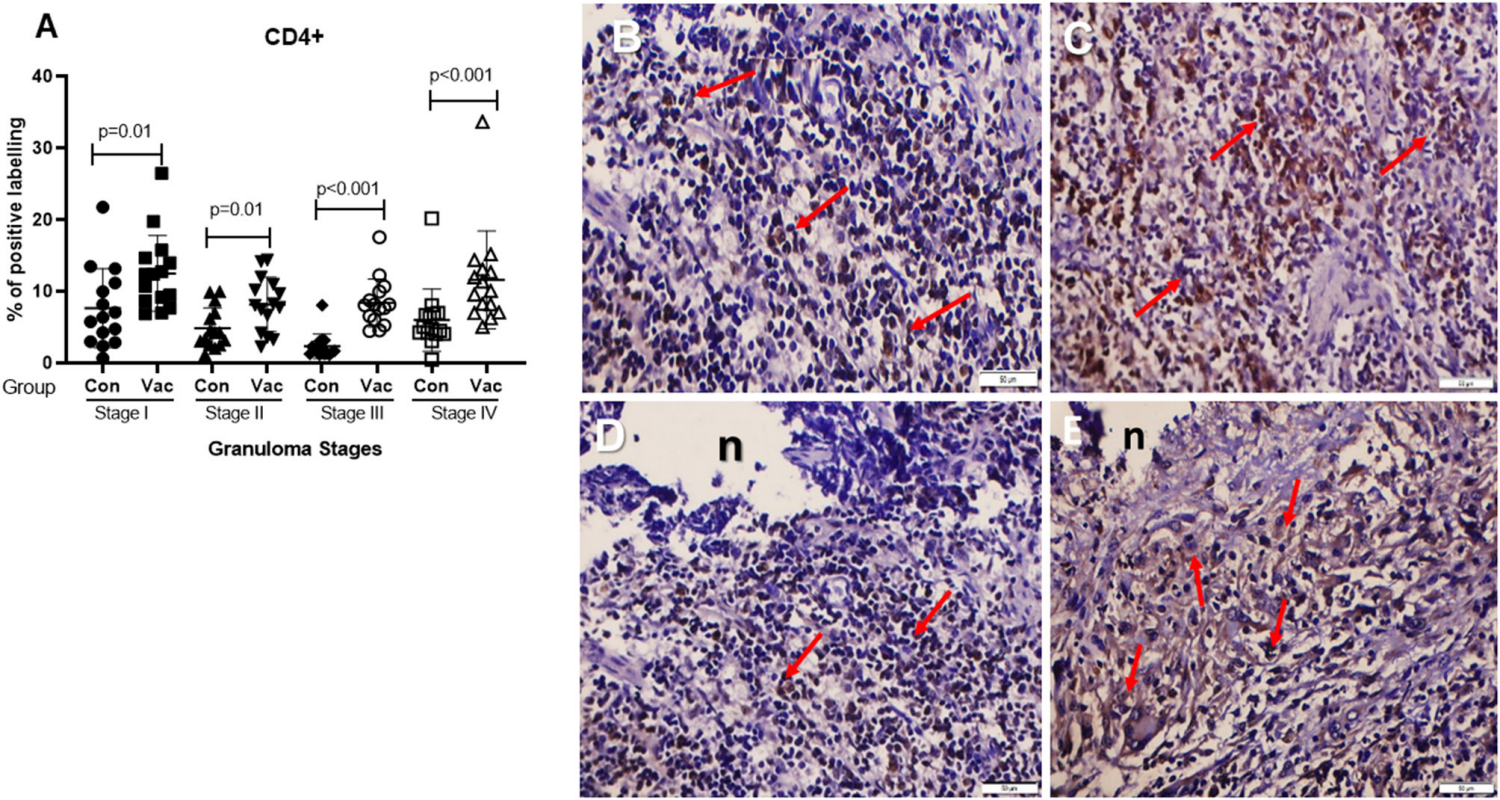
Immunolabeling for CD4^+^ cells (ABC IHC stain, DAB brown chromogen). **(A)** Comparison of CD4^+^ cell distribution in different granuloma stages of BCG vaccinated (Vac, vaccinated) and non-vaccinated (Con, control) calves. **(B)** Stage I granuloma from non-vaccinated calves, showing stained CD4^+^ cells (arrows). **(C)** Stage I granuloma from BCG vaccinated calf showing more CD4^+^ cells (arrows). **(D)** Stage IV granuloma from non-vaccinated calf showing stained CD4^+^ cells (arrows). **(E)** Stage IV granuloma from BCG vaccinated calf showing stained CD4^+^ cells (arrows). Bar equals 50μm in **(B–E)**. n, necrotic core.

**Figure 2:**
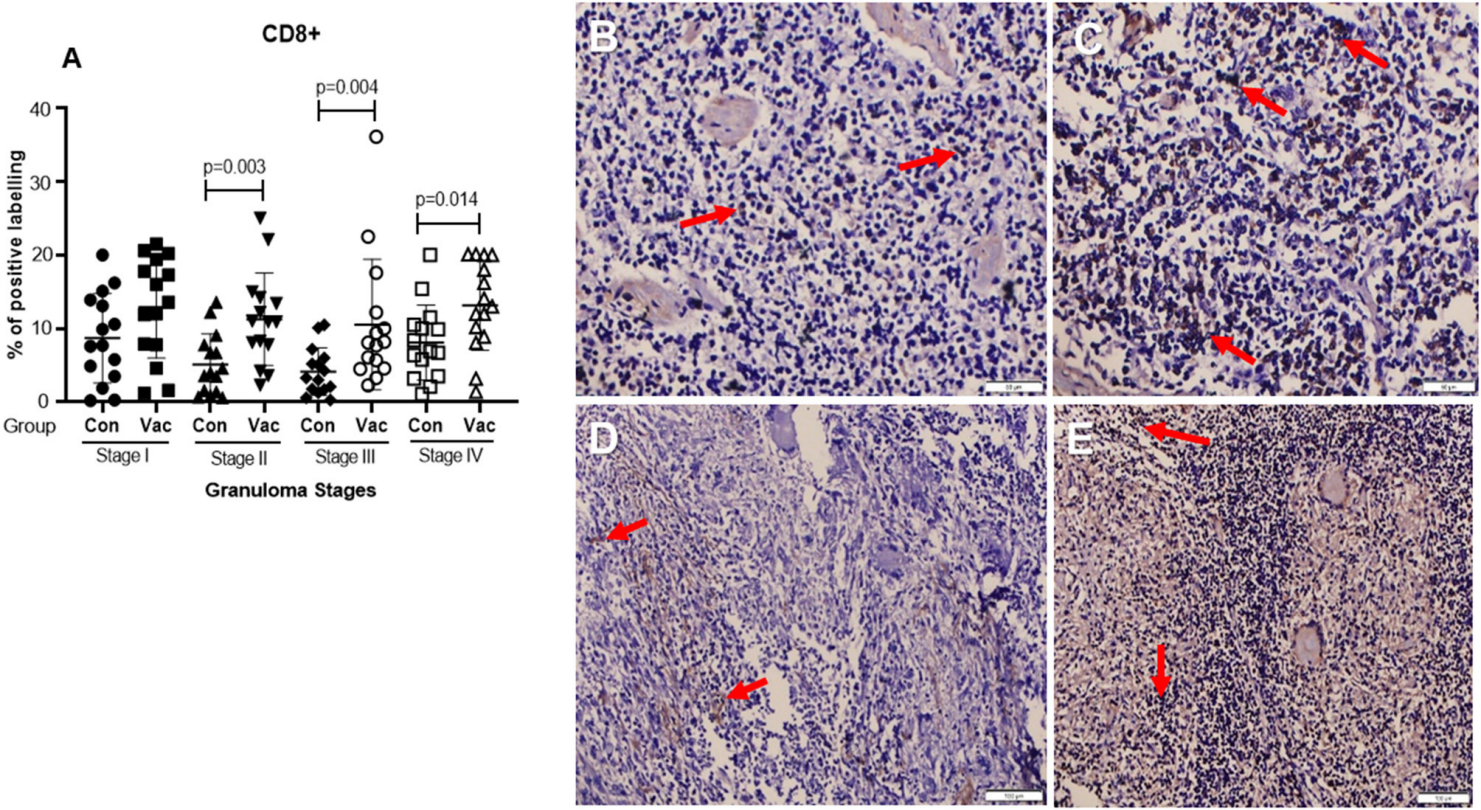
Immunolabelling for CD8^+^ cells (ABC IHC stain, DAB brown chromogen). **(A)** Comparison of CD8^+^ cell distribution in different granuloma stages of BCG vaccinated (Vac) and non-vaccinated (Con) calves. **(B)** Stage I granuloma from non-vaccinated calf showing very few stained CD8^+^ cells (arrows). **(C)** Stage I granuloma from BCG vaccinated calf showing stained CD8^+^ cells (arrows). **(D)** Stage III granuloma from non-vaccinated calf showing few stained CD8^+^ cells (arrows). **(E)** Stage III granuloma from vaccinated calf showing abundance of CD8^+^ cells (arrows). Bar equals 50μm in **(B,C)** and 100μm in **(D,E)**.

**Figure 3:**
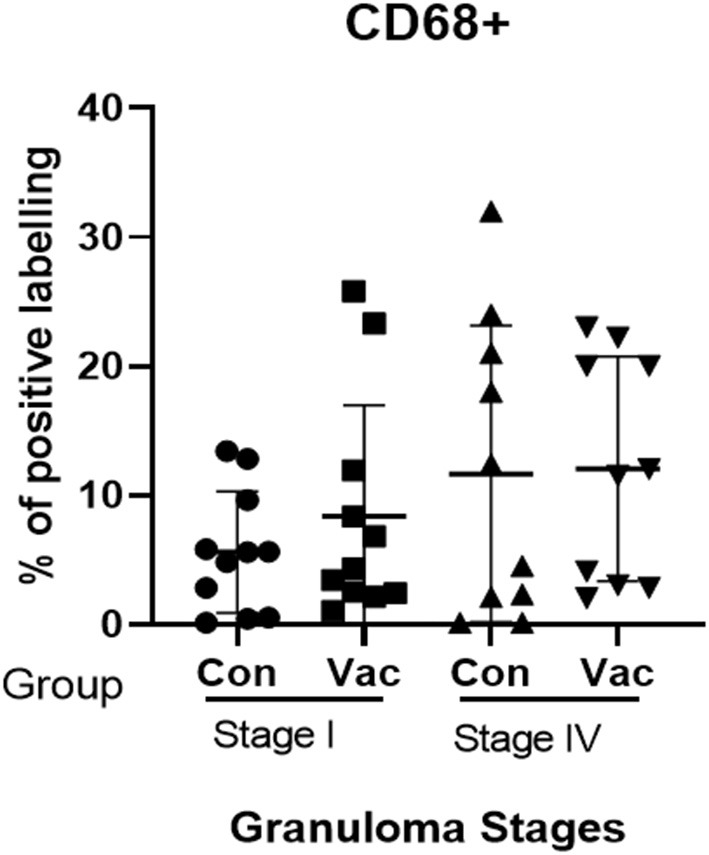
Immunolabelling for CD68^+^ cells. Comparison of CD68^+^ cell distribution in granuloma stages I and IV of BCG vaccinated (Vac) and non-vaccinated (Con) calves.

**Figure 4:**
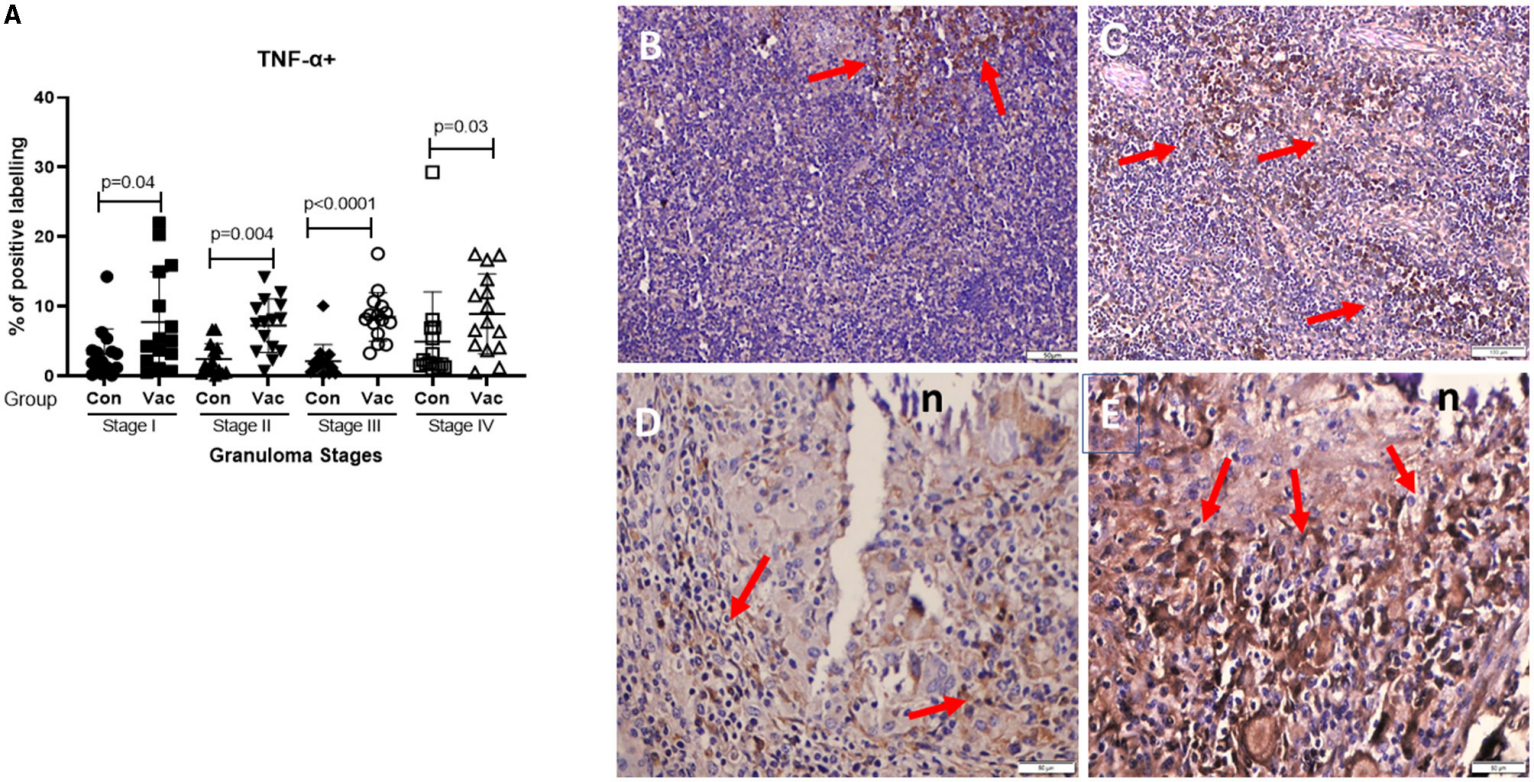
Immunolabelling for TNF-α^+^ (ABC IHC stain, DAB brown chromogen). **(A)** Comparison of TNF-α^+^ distribution in different granuloma stages of BCG vaccinated (Vac) and non-vaccinated (Con) calves. **(B)** Stage I granuloma from non-vaccinated calf with few number of TNF-α stained macrophages (arrows), **(C)** Stage I from BCG vaccinated calf with more positively stained macrophages (arrows). **(D)** Stage IV granuloma from non-vaccinated calf with moderate number of positive cells (arrows). **(E)** Stage IV granuloma from BCG vaccinated calf with abundance of positively stained cells (arrows). Bar equals 100μm in **(B,C)**, and 50μm in **(D,E)**. n, necrotic core.

**Figure 5:**
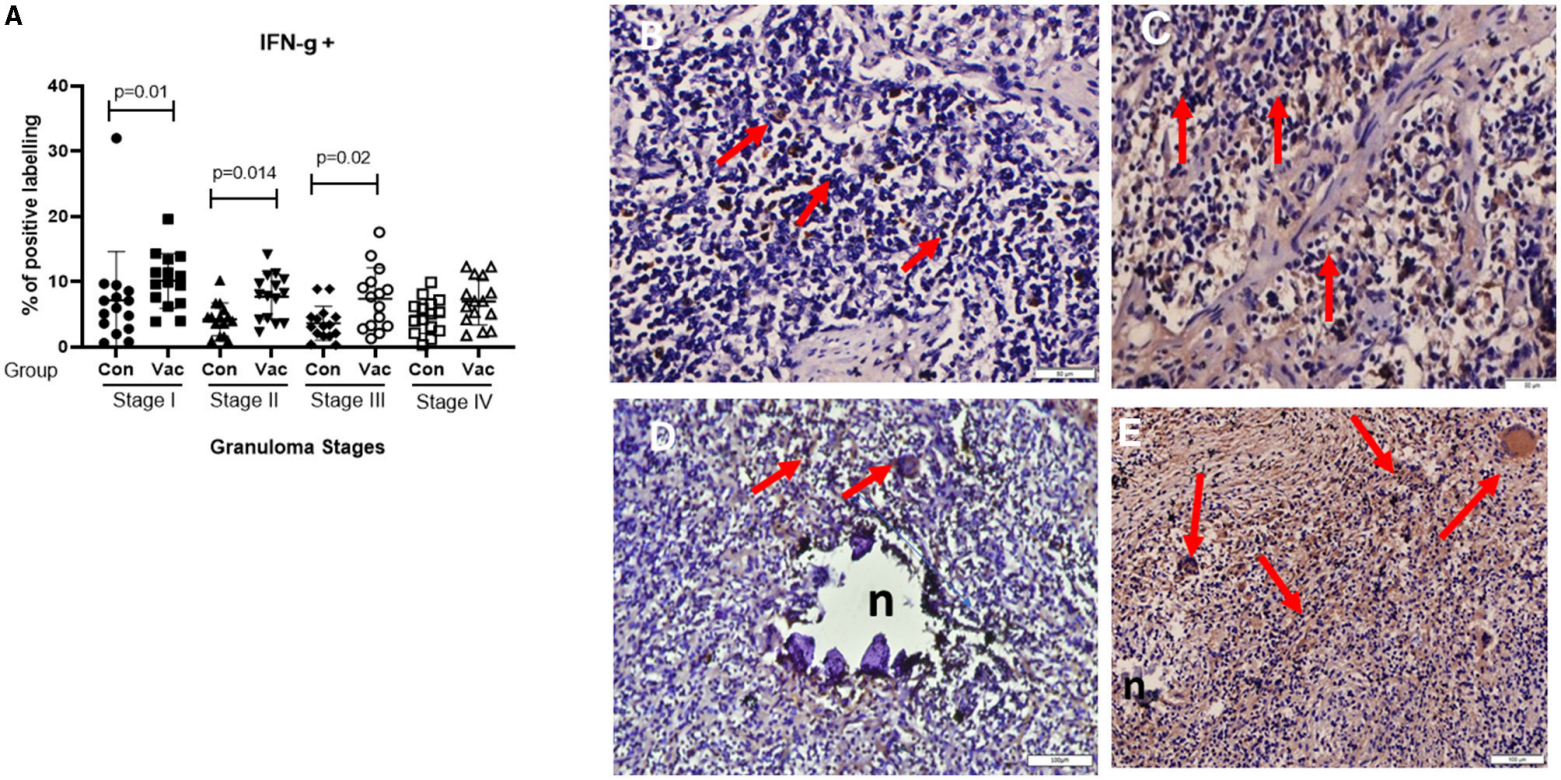
Immunolabelling for IFN-γ^+^. **(A)** Comparison of IFN-γ^+^ cell distribution in different granuloma stages of BCG vaccinated (Vac) and non-vaccinated (Con) calves. **(B)** Stage I granuloma from non-vaccinated calf with some IFN-γ^+^ positively stained cells (arrows). **(C)** Stage I granuloma from BCG vaccinated calf has more lymphocytes stained for IFN-γ^+^ (arrows). **(D)** Stage IV granuloma showing IFN-γ^+^ stained cells from non-vaccinated calf (arrows). **(E)** Stage IV granuloma showing IFN-γ^+^ stained cells from BCG vaccinated calf (arrows). 50μm for **(B,C)**, Bar equals 100μm in **(D,E)**.

**Figure 6:**
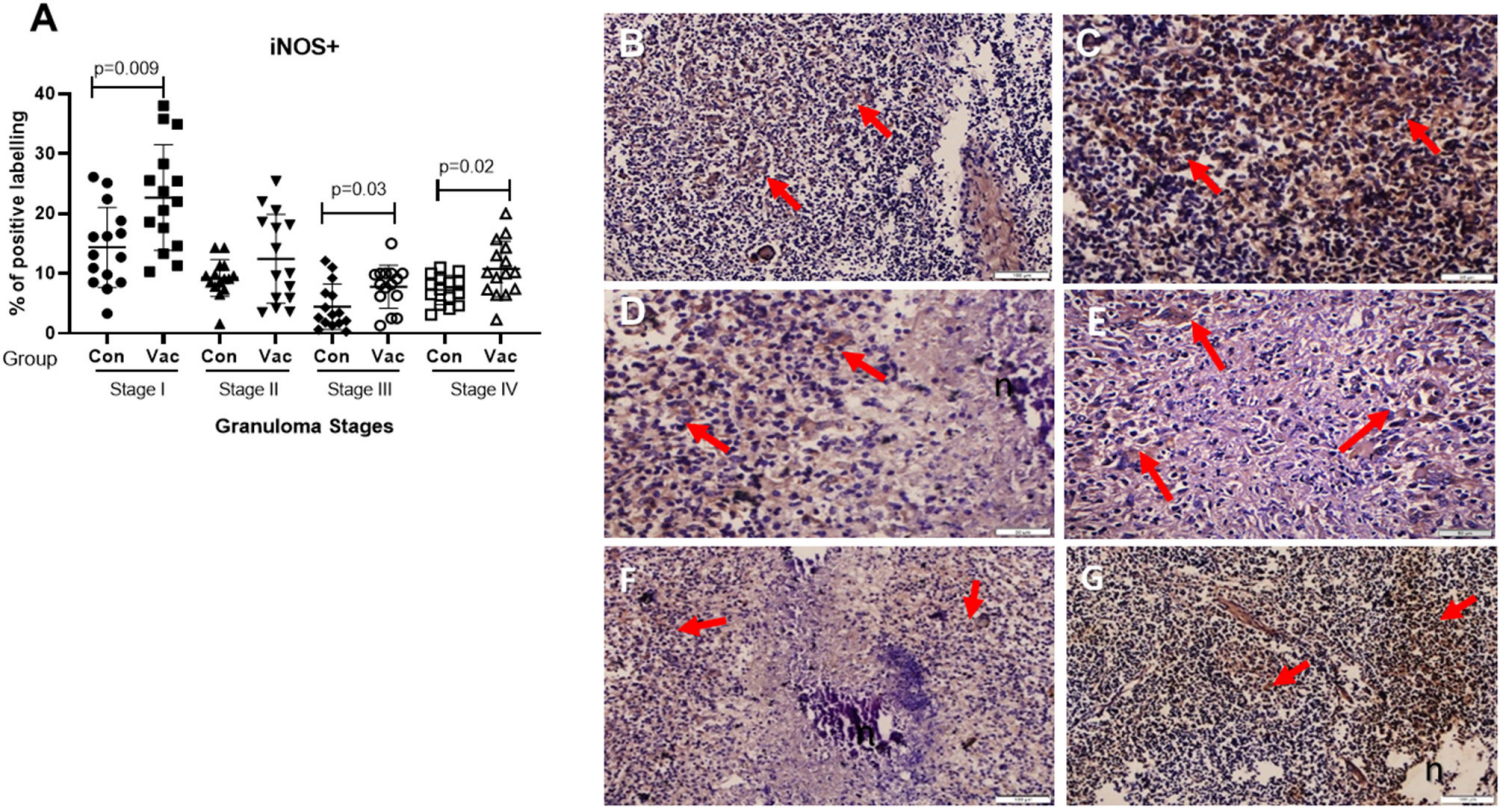
Immunolabelling for iNOS^+^. **(A)** Comparison of iNOS^+^ cell distribution in different granuloma stages of BCG vaccinated (Vac) and non-vaccinated (Con) calves. **(B)** Stage I granuloma from non-vaccinated calf with some iNOS^+^ positively stained cells (arrows). **(C)** Stage I granuloma from BCG vaccinated calf, many cells stained for iNOS^+^ (arrows). **(D)** Stage III granuloma from non-vaccinated calf showing stained cells with iNOS^+^ (arrows). **(E)** Stage III granuloma showing cells stained positively with iNOS^+^ from BCG vaccinated calf (arrows). **(F)** Stage IV granuloma from non-vaccinated calf showing cells stained with iNOS^+^ (arrows). **(G)** Stage IV granuloma from BCG vaccinated calf showing many cells stained with iNOS^+^ (arrows). Bar equals in **(B,C)** 50 μm, Bar equals 100μm **(D–G)**. n, necrotic core.

IHC was used to detect CD68+ macrophages, CD4+ and CD8+ T cells, TNF-α, IFN-γ, and iNOS within tissue sections. The responses of these immunological markers were compared between controls and vaccinated calves and further stratified by different granuloma stages. The positive labeling was expressed as a fraction of the total area examined in each slide. The following results of cellular and cytokine responses were quantified:

**CD4+ cells**. Immunohistochemical staining of CD4 demonstrated an increase in the number of stained cells in the BCG vaccinated calves compared to non-vaccinated calves in different stages of granuloma. The fraction of immunolabeled CD4^+^ cells among vaccinated calves was significantly higher (*P* < 0.05) in all four granuloma stages when compared with the non-vaccinated group (Figure 1).
**CD8+ cells**. Similarly, immunohistochemical staining of CD8 showed higher numbers of CD8+ cells in BCG vaccinated calves than in non-vaccinated calves and the difference was significant (*P* < 0.05) between the groups in stages II-IV (Figure 2).
**Macrophages (CD68+)**. The immunohistochemical staining revealed no significant differences in CD68+ cell distribution in the different granuloma stages of the BCG vaccinated and non-vaccinated calves (Figure 3). The vaccinated group indicated a higher fraction of immune-labeled cells than the non-vaccinated group for stage I granulomas, but there was no statistically significant difference between the two groups (*P* > 0.05).
**TNF-α^+^**. The TNF-α^+^ immunohistochemical staining showed higher TNF-α^+^ response in BCG vaccinated as compared to the level of response in non-vaccinated calves (Figure 4), and that was the case in all four stages of granuloma (*P* < 0.05).
**IFN-γ**. Immunohistochemical staining for IFN-**γ** indicated an increased expression of IFN-γ in BCG vaccinated compared to non-vaccinated calves in all four granuloma stages, however, only in stage I-III were this increase statistically significant between the groups (*P* < 0.05) (Figure 5).
**iNOS**^**+**^. Positive staining for iNOS^+^ was also present at all stages of granulomas in both BCG vaccinated and non-vaccinated calves, and the analysis further demonstrated a statistically higher expression in BCG vaccinated calves at stage I, stage III, and stage IV (Figure 6).

## Discussion

In the present study, immunological responses were evaluated at local granuloma level in 15 BCG-vaccinated and 16 non-vaccinated calves that had been naturally infected with *M. bovis* and that showed gross visible lesions at the *post-mortem* examination. On the other hand, nine calves (seven from the vaccinated and two from the non-vaccinated group) had either not become infected or had no visible lesions. In the present study, more stage IV granuloma was observed in the mesenteric lymph node of vaccinated calves than in control calves. This observation can be considered as misleading, as such difference was more likely associated with the sampling procedure and preparation of tissue sections of the two groups of calves. As observed during post mortem examination, gross lesions were detected in the mesenteric lymph nodes of one vaccinated and one control calves. In both calves, the pathology score was 3, which is considered as the most severe for the lymph node score. This indicates that the severity of the gross pathology was similar in both the vaccinated and non-vaccinated calves. However, in microscopic pathological comparison, stage IV (the most severe microscopic pathology) was observed in the mesenteric lymph node of the vaccinated calf than in the mesenteric lymph node of the control calf. As the two calves had similar severity of gross pathology, the difference in microscopic pathology between the two calves is mainly attributed to the difference in the sampling of the two lymph nodes and or the difference in the sections of the two lymph nodes used for the evaluation of the microscopic lesions. In the case of the vaccinated calf, the part of the mesenteric lymph node that was sampled and examined was severely affected while in the case of the non-vaccinated calf, the part of the mesenteric lymph node that was sampled was less severely affected and could the peripheral part of the lymph node. But in general, it was observed that both gross and microscopic pathology were relatively less severe in vaccinated calves than in non-vaccinated calves. Substantiating this observation, a recently conducted meta-analysis suggested that BCG vaccination may help in accelerate control of bTB in endemic settings by inducing immunity against *M. bovis* and also limiting the progress TB lesion at the site of infection (33).

In the current study, the immunohistochemical staining of CD68, CD4, CD8, IFN-γ, TNF-α, and iNOS were evaluated and compared in BCG-vaccinated and non-vaccinated calves. The presence of these immunological markers was also compared between the different stages of granulomas using IHC staining on histological sections of granulomas from lymph nodes and lungs. The results showed stronger responses of CD4+ and CD8+ T cells in granulomas of the BCG vaccinated calves when compared to those from the unvaccinated group. This observation is consistent with the results in a study conducted by Hope et al. (34) who also reported higher induction of CD4+ and CD8+ T cell responses in BCG-vaccinated Holstein cattle compared to non-vaccinated (34). Studies have shown that the majority of CD8+ T cells in the local immunity outside the peripheral circulation are γδ T cells (35). In this regard, it is important to look into the expressions of γδ T cells at local lesion sites during natural *M. bovis* infection.

Phagocytic cells such as macrophages and dendritic cells, and T cells including CD4+, CD8+, and γδ T cells play important roles in controlling *M. bovis* infection in cattle (36). The observation of responses of these cells in bTB granulomas in the lymph nodes of BCG-vaccinated calves could suggest the significance of BCG in protecting against bTB (37). In the present study, the expression levels of CD68+ macrophages in the granulomas of vaccinated and non-vaccinated calves were not significantly different. On the contrary, Salguero et al. (38) reported a significant reduction in the expression of CD68+ macrophages in granulomas of BCG-vaccinated Holstein calves as compared to its expression in non-vaccinated animals, after being experimentally inoculated intratracheally (38). On the other hand, a study conducted by Tulu et al. (39) on *M. bovis* naturally infected cross breed cows showed a significant increase in the immune-labeling of CD68+ macrophages as the level of granuloma increases from stage I to IV in culture positive animals as compared to culture negative animals. However, at the early stages of granuloma formation, the immune-labeling of CD68+ macrophages was significantly higher for *M. bovis* culture negative cows as compared to culture positive animals, which was explained in terms of the role of CD68+ macrophages geared toward the protection and elimination mycobacteria (39).

Furthermore, the result of the present study showed that the expressions of IFN-γ, TNF-α, and iNOS were stronger in granulomas of vaccinated calves than in non-vaccinated calves. The protective role of IFN-γ in the response to *M. bovis* infection in cattle is well-established (40). Previous experimental infection reported that IFN-γ, TNF-α, and iNOS showed increased expressions among the lymph node granulomas of BCG vaccinated cattle compared to non-vaccinated (38). Additionally, TNF-α is known to act in conjunction with IFN-γ to induce the release of reactive oxygen and nitrogen in infected macrophages, which are required for the killing of intracellular bacilli (41). On the other hand, such release can become toxic at high concentrations through an increase in necrosis and tissue damage (42). Previous studies have indicated that the macrophage and iNOS activity are more intense and sustained along the granuloma development (43, 44).

### Limitation of the Study

The comparison of the microscopic pathology and immunological markers between the vaccinated and non-vaccinated calves were based on the examinations of one or few sections of tissues. The results of examinations of a single section or few sections does not give realistic comparison. Several sections should have been prepared and examined the comparisons of microscopic pathology and immunological markers between the vaccinated and control calves. The other limitation of this study is the consideration of only a few markers for comparison between the two groups of calves. Additional cell and cytokine, or chemokine markers could have been included in the study. In addition to the above two limitations, the use zinc solution fixed tissues for the histopathological examination and classification of granuloma. Tissue fixation with zinc solution is recommended for the investigation of CD4+ and CD8+ cells markers, but histopathological examination of tissues fixed with zinc solution is laborious and difficult to delineate the different stages of granuloma.

## Conclusion

BTB continues to be a major animal and public health problem worldwide and new tools are required for the control of this disease, primarily in cattle. In the present study, vaccination of calves with BCG induced strong responses at the granulomas of CD4+ and CD8+ T cells, and the two cytokines TNF-α and IFN-γ as well as for the chemical mediator iNOS. These observations could suggest the potential of BCG vaccination in containing the spread of *M. bovis* and in reducing the severity of gross pathology at the primary sites of infection.

## Data Availability Statement

The raw data supporting the conclusions of this article will be made available by the authors, without undue reservation.

## Ethics Statement

The animal study was reviewed and approved by Institutional Review Board (IRB) of Aklilu Lemma Institute of Pathobiology, Addis Ababa University (Reference number IRB/ALIPB/013/1017/18).

## Author Contributions

AS and GA conceived the study. AS and BB conducted the field and laboratory aspects of the study. FS and GA supervised and lead the study. AS, BT, and FS analyzed the result of the study and interpreted the result. AS, BT, and BG drafted the manuscript while FS, SB, and GA edited the manuscript. All authors reviewed the final draft and agreed with its content and conclusions.

## Funding

This study was funded by the Biotechnology and Biological Sciences Research Council (BBSRC), the Department for International Development, the Economic & Social Research Council, the Medical Research Council, the Natural Environment Research Council and the Defense Science & Technology Laboratory, under the Zoonoses and Emerging Livestock Systems (ZELS) program, ref: BB/L018977/1. SB was also partly funded by the Department for Environment, Food & Rural Affairs, United Kingdom, for this study, ref: TBSE3294.

## Conflict of Interest

The authors declare that the research was conducted in the absence of any commercial or financial relationships that could be construed as a potential conflict of interest.

## Publisher's Note

All claims expressed in this article are solely those of the authors and do not necessarily represent those of their affiliated organizations, or those of the publisher, the editors and the reviewers. Any product that may be evaluated in this article, or claim that may be made by its manufacturer, is not guaranteed or endorsed by the publisher.
