# Supplementary material for: Cellular and Cytokine Responses in Lymph Node Granulomas of Bacillus Calmette Guérin (BCG)-Vaccinated and Non-vaccinated Cross-Breed Calves Naturally Infected With Mycobacterium bovis
Source: Front Vet Sci. 2021 Sep 16;8:698800. doi: 10.3389/fvets.2021.698800 (PMC8483244; doi:10.3389/fvets.2021.698800)
Supplement: Supplementary file 1 [file Table_1.docx]

Supplementary Table S1: Distribution of granulomas of different developmental stages (I-IV) of BCG vaccinated and non-vaccinated (control) calves. Data collected in Bayissa et al. (submitted for publication)

| **Lymph node (LN)/Tissue** | **Group** | **Stages of granuloma** | | | |
| --- | --- | --- | --- | --- | --- |
|  |  | **I** | **II** | **III** | **IV** |
| Head and neck LN* | Control | 22 | 1 | 3 | 13 |
|  | BCG Vaccinated | 21 | 0 | 0 | 0 |
| Thoracic LN* | Control | 83 | 25 | 18 | 94 |
|  | BCG Vaccinated | 90 | 27 | 16 | 47 |
| Abdominal LN* | Control | 5 | 2 | 0 | 0 |
|  | BCG Vaccinated | 5 | 0 | 1 | 13 |
| Lung tissue | Control | 43 | 4 | 4 | 15 |
|  | BCG Vaccinated | 0 | 3 | 3 | 0 |
| **TOTAL*** | Control | 153 | 32 | 25 | 122 |
|  | BCG Vaccinated | 116 | 30 | 20 | 60 |

1. *P<0.05 (chi-square for trend) between the two groups
